# The Cultivable Surface Microbiota of the Brown Alga *Ascophyllum nodosum* is Enriched in Macroalgal-Polysaccharide-Degrading Bacteria

**DOI:** 10.3389/fmicb.2015.01487

**Published:** 2015-12-24

**Authors:** Marjolaine Martin, Tristan Barbeyron, Renee Martin, Daniel Portetelle, Gurvan Michel, Micheline Vandenbol

**Affiliations:** ^1^Microbiology and Genomics Unit, Gembloux Agro-Bio Tech, University of LiègeGembloux, Belgium; ^2^Sorbonne Université, UPMC, Centre National de la Recherche Scientifique, UMR 8227, Integrative Biology of Marine ModelsRoscoff, France

**Keywords:** *Flavobacteriia*, *Gammaproteobacteria*, macroalgae, agarase, carrageenase, alginate lyase, *Ascophyllum nodosum*, algal polysaccharidase

## Abstract

Bacteria degrading algal polysaccharides are key players in the global carbon cycle and in algal biomass recycling. Yet the water column, which has been studied largely by metagenomic approaches, is poor in such bacteria and their algal-polysaccharide-degrading enzymes. Even more surprisingly, the few published studies on seaweed-associated microbiomes have revealed low abundances of such bacteria and their specific enzymes. However, as macroalgal cell-wall polysaccharides do not accumulate in nature, these bacteria and their unique polysaccharidases must not be that uncommon. We, therefore, looked at the polysaccharide-degrading activity of the cultivable bacterial subpopulation associated with *Ascophyllum nodosum*. From *A. nodosum* triplicates, 324 bacteria were isolated and taxonomically identified. Out of these isolates, 78 (~25%) were found to act on at least one tested algal polysaccharide (agar, ι- or κ-carrageenan, or alginate). The isolates “active” on algal-polysaccharides belong to 11 genera: *Cellulophaga, Maribacter, Algibacter*, and *Zobellia* in the class *Flavobacteriia* (41) and *Pseudoalteromonas, Vibrio, Cobetia, Shewanella, Colwellia, Marinomonas*, and *Paraglaceciola* in the class *Gammaproteobacteria* (37). A major part represents likely novel species. Different proportions of bacterial phyla and classes were observed between the isolated cultivable subpopulation and the total microbial community previously identified on other brown algae. Here, *Bacteroidetes* and *Gammaproteobacteria* were found to be the most abundant and some phyla (as *Planctomycetes* and *Cyanobacteria*) frequently encountered on brown algae weren't identified. At a lower taxonomic level, twelve genera, well-known to be associated with algae (with the exception for *Colwellia*), were consistently found on all three *A. nosodum* samples. Even more interesting, 9 of the 11 above mentioned genera containing polysaccharolytic isolates were predominant in this common core. The cultivable fraction of the bacterial community associated with *A. nodosum* is, thus, significantly enriched in macroalgal-polysaccharide-degrading bacteria and these bacteria seem important for the seaweed holobiont even though they are under-represented in alga-associated microbiome studies.

## Introduction

Polysaccharide-degrading bacteria are key players in the global carbon cycle and have an increasing importance in biotechnology and biomass utilization. In terrestrial environments, plants host a remarkable diversity of microbes, representing a continuum of symbioses ranging from mutualism to commensalism to pathogenic behavior (Bulgarelli et al., 2013). Among these microbes, numerous bacteria possess polysaccharidases involved in the utilization of plant cell wall polymers (Gibson et al., 2011). Soils also constitute an important reservoir of hemicelluloytic microorganisms, which carry on the mineralization of plant polysaccharides (DeAngelis et al., 2010; Leung et al., 2015). In coastal ecosystems, macroalgae are crucial primary producers, constituting a huge biomass and playing an ecological role analogous to that of plants in terrestrial environments. Brown, green, and red macroalgae constitute three independent eukaryotic lineages, and their cell walls contain highly diverse polysaccharides, notably sulfated polysaccharides having no equivalent in land plants (Popper et al., 2011). By comparison with what is known about biomass recycling in terrestrial environments, knowledge of bacterial recycling of macroalgal biomass trails far behind. Nonetheless, the discovery of glycoside hydrolases (GH) and polysaccharide lyases (PL) targeting macroalgal polysaccharides has accelerated in the last 15 years (Michel and Czjzek, 2013). These particular enzymes were found to constitute either new subfamilies within known Carbohydrate-Active enZYmes (CAZy) families [e.g., κ-carrageenases and β-porphyranases in the GH16 family (Michel et al., 2001; Hehemann et al., 2010)] or new CAZy families, such as ι-carrageenases (GH82; Barbeyron et al., 2000), α-agarases (GH96; Flament et al., 2007), and fucoidanases (GH107; Colin et al., 2006).

The majority of these enzymes have been isolated from seaweed-associated bacteria (Martin et al., 2014b). MAPD bacteria have been found essentially in two classes: (i) *Gammaproteobacteria*, in the phylum *Proteobacteria* (e.g., *Agarivorans albus, Alteromonas agarilytica, Pseudoalteromonas carrageenovora, Vibrio* sp. PO-303) and (ii) *Flavobacteriia*, in the phylum *Bacteroidetes* (e.g., *Cellulophaga lytica, Flammeovirga yaeyamensis, Mariniflexile fucanivorans, Zobellia galactanivorans*; for reviews see Michel and Czjzek, 2013; Martin et al., 2014b). Despite the identification of these bacteria, most MAPD enzymes currently constitute very small protein families: e.g., GH16 κ-carrageenases (six proteins); GH16 β-agarases (41), GH16 β-porphyranases (7), GH82 ι-carrageenases (19), GH96 α-agarases (4), GH107 fucoidanases (4), and GH118 β-agarases (8) (http://www.cazy.org; Lombard et al., 2014). However, as macroalgal cell-wall polysaccharides do not accumulate in nature, MAPD bacteria and their unique polysaccharidases must not be that uncommon.

The emerging metagenomic exploration of marine environments (Venter et al., 2004; Yooseph et al., 2007; Gómez-Pereira et al., 2012) offers the promise of increasing the discovery of novel enzymes, either through data mining or through activity screening of metagenomic libraries (Lee et al., 2010; Ferrer et al., 2015). Marine metagenomics has yielded disappointing results, however, as regards MAPD enzymes. For instance, in the entire Global Ocean Survey (GOS) dataset (Yooseph et al., 2007), there is not a single κ-carrageenase gene, only one ι-carrageenase gene, 30 coding DNA sequences (CDSs) for α-1,3-(3,6-anhydro)-L-galactosidase (the terminal enzyme of agar degradation, AhgA, GH117) and five times fewer CDSs for β-agarase and β-porphyranase, and seventeen PL17 alginate lyases (Hehemann et al., 2010; Rebuffet et al., 2011; Neumann et al., 2015). Clearly, this relative lack of success in finding MAPD enzymes in the water column suggests that bacterioplanktons rarely possess such enzymes. However, as most MAPD enzymes have been isolated from seaweed-associated bacteria, one should think that seaweed-associated microbiome datasets contain plenty of these specific enzymes. In fact, in the only two existing studies sequencing the microbiota associated with macroalgae (with *Delisea pulchra* and *Ulva australis*), no polysaccharidase gene is discussed (Fernandes et al., 2012), or only genes related to alginate metabolism are briefly mentioned (Burke et al., 2011). Unfortunately, we couldn't find out if MAPD enzymes were present in the these macroalgae associated microbiota, as the metagenomic datasets of these two studies were deposited on the CAMERA database (http://camera.calit2.net/) that has shut down and its successor, iMicrobe (http://data.imicrobe.us/), does not contain these datasets. Also, a transcriptomic analysis of the microbiota of the red macroalga *Laurencia dendroidea* has been performed by RNA-Seq, but again, algal-polysaccharidase genes were either overlooked or absent from the dataset (de Oliveira et al., 2012). The metatranscriptomic datasets associated to *L. dendroidea* is publicly available on the MG-Rast database (de Oliveira et al., 2012). We searched in the protein files corresponding to these datasets sequence identities with the characterized MAPD enzymes (Michel and Czjzek, 2013; Martin et al., 2014b) using BlastP (NCBI), but we did not find any homologous protein (data not shown). Concerning functional metagenomic analyses of seaweed-associated microbiota, only two studies have previously been carried out. On the one hand, the microbial community associated with *Ulva australis* was searched for antibacterial proteins (Yung et al., 2011). On the other hand, our group has recently screened the microbiota of the brown alga *Ascophyllum nodosum* for hydrolytic enzymes, especially glycoside hydrolases. As proof of feasibility, we used established activity tests and identified 13 esterases from *Alphaprotobacteria* and *Gammaproteobacteria* members, a GH3 β-glucosidase from an *alphaprotobacterium*, and a GH5 cellulase from a *gammaproteobacterium* (Martin et al., 2014a). Taking advantage of the ability of κ- and ι-carrageenans, agar, and alginate to form gels, we subsequently developed activity tests for screening a second *A. nodusum* metagenomic library for the corresponding polysaccharidases. The quality of this metagenomic library was validated and several esterases and β-glucosidases were again isolated. Surprisingly, however, we did not find any MAPD enzymes (unpublished results).

This disappointing result and the almost absence of MAPD enzymes from other metagenomic studies on seaweed-associated microbiota raised several questions. Are MAPD bacteria sufficiently abundant at the surface of macroalgae? Are metagenomic analyses of entire seaweed-associated microbiota the most adequate approach to finding MAPD enzymes? As a first step toward answering such questions, we have chosen to focus on the cultivable subpopulation of bacteria associated with *A. nodosum* and to establish a direct link with the catabolic capacities of these microorganisms. For this we have isolated, on a marine medium, 324 cultivable bacteria from triplicate *A. nodosum* samples. We have taxonomically identified these bacterial isolates and have screened them for agarases, κ-carrageenases, ι-carrageenases, and alginate lyases. Bacterial isolates found to display at least one such activity were further investigated.

## Materials and methods

### Phylogenetic and statistical analyses of the bacterial communities isolated from the three *Ascophyllum nodosum* samples

Healthy *A. nodosum* plants were collected in triplicate from the foreshore in Roscoff (Brittany, France) at the end of March, 2014. The samples were rinsed three times with sterile seawater to remove loosely attached microorganisms and used immediately for microbial isolation. Cultivable microorganisms were isolated by swabbing algal surfaces with sterile cotton tips as in our previous functional metagenomics study (Martin et al., 2014a) and then inoculating plates of marine medium (Marine Agar, Difco). One plate per sample was inoculated and for each sample we used 20 cm of thallus. Plates were left at room temperature. After 4 days, 108 isolated colonies, representative of our total isolated colonies, were recovered at random from each plate (324 colonies in all) in 96-well plates with marine medium (Marine Broth, Difco). Glycerol (final concentration 20%) was added to the liquid bacterial cultures in the 96-well plates and conserved at −80°C.

**Figure 1:**
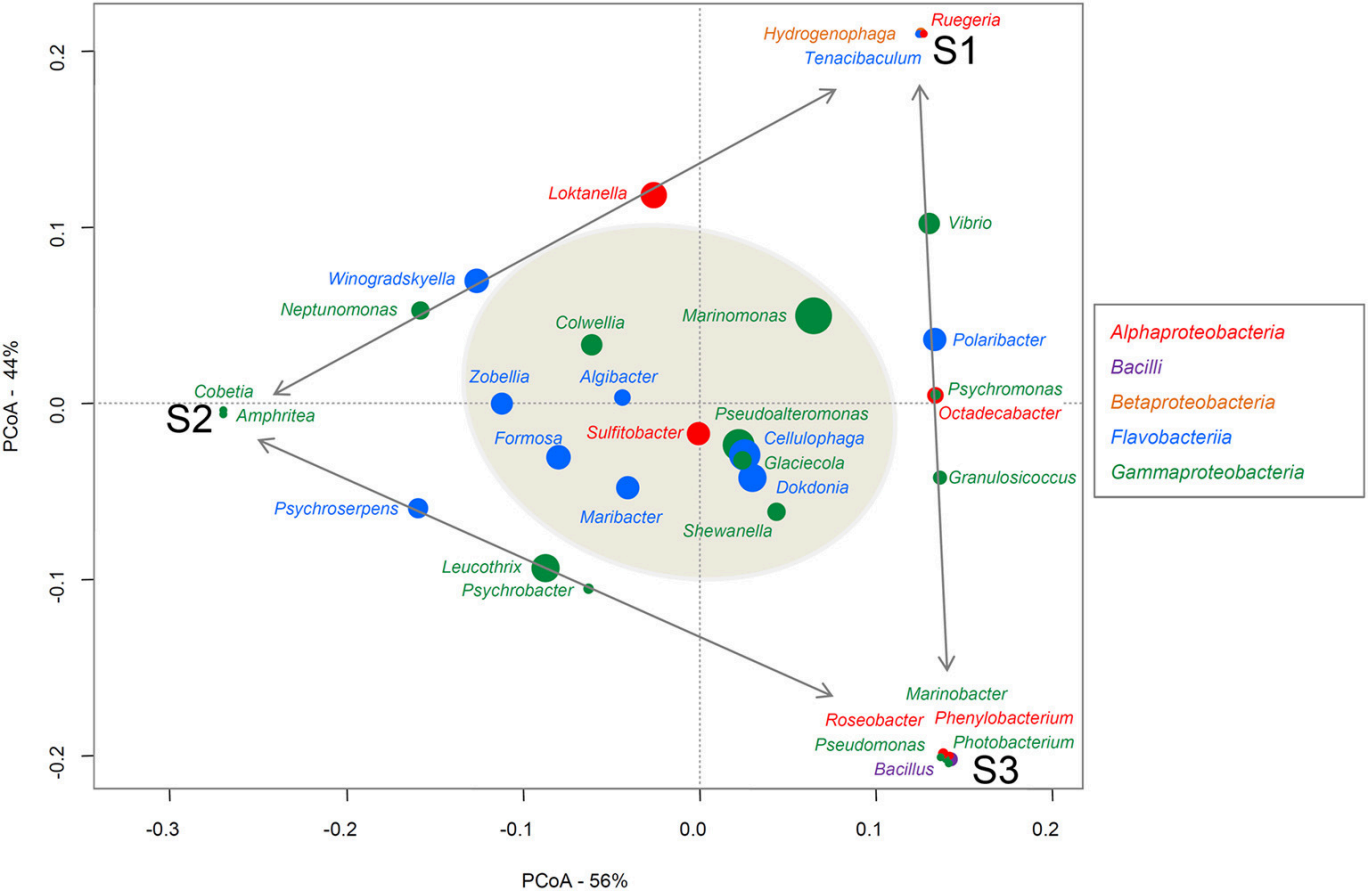
**PCoA of the genera found on the three alga samples**. Bacterial classes are indicated in different colors. The size of each spot is proportional to the relative abundance of the genus indicated above or next to it. The closer a spot is to a sample name, the more abundant this genus is in that sample. The Genera common to just to samples are aligned along the gray arrows. The genera identified on the three samples (i.e., the common core) are in the gray shadowed circle.

To assign taxa to the 324 bacteria isolated from the three alga samples, the genus-specific V3-V4 region of the 16S rRNA (~400 bp) was amplified and sequenced. First, 5 μl stock of bacteria in glycerol was mixed with 10 μl PCR water and heated at 95°C for 10 min to lyse the bacteria. Then 5 μl bacterial lysate was added to the PCR mix [200 μM dNTP, 0.4 μM primer F (AllBactF 5′-TCCTACGGGAGGCAGCAGT-3′), 0.4 μM primer R (AllBactR 5′-GGACTACCAGGGTATCTAATCCTGTT-3′; Nadkarni et al., 2002; Stroobants et al., 2014), PCR buffer 1x, Taq DNA polymerase, final volume 50 μl] for the PCR [94°C- 7′, 35 × (95°C-30″), 60°C-30″, 65°C -1′, 68°C-7′]. Primers were produced at Eurogentec (BE) and PCR reagents were provided by Roche. PCR were realized in 96-well plates and amplicons were sequenced by Sanger Sequencing at GATC using the AllBactF forward primer (GATC Biotech, Germany). Sequences were aligned with sequences from the GenBank_Bacteria and SILVA SSU databases (*E*-value threshold 0.01) and each bacterium was taxonomically identified at the genus, family, order, class, and phylum level. Averages and percentage ranges of the resulting query coverages and identity percentages have been summarized for each sample and for the total isolated microbiota in the Supplementary Material (Table S1). A Principal Coordinates Analyses (PCoA) was realized with the relative abundance of each genus in each sample to represent the differences in composition of the three samples (Figure 1). Relative abundances of the genera were log transformed and Bray Curtis matrix distance was used. For each genus, a weight average was calculated based on the abundance of this genus in each sample and the axes-coordinates of the sample, in order to reveal affinities between a genus and one, two or the three samples. The PCoA plot was performed using the VEGAN Package (Dixon, 2003; Oksanen et al., 2008) implemented in the R statistical software on the bacterial genera identified on the three algae samples (Figure 1).

### Isolation of bacteria with polysaccharidase activities

The 324 isolated colonies were grown at 22°C for 24 h in 96-microwell plates containing marine broth and then plated on marine broth containing 1.5% agar, 1% κ-carrageenan, 2% iota-carrageenan, or on modified Zobell medium (24.7 g/l NaCl, 6.3 g/l MgSO_4_, 0.7 g/l KCl, 5 g/l tryptone, 1 g/l yeast extract) with 5% alginate salts and 40 mM MgCl_2_. All chemicals were purchased from Sigma-Aldrich. After 48 h to 1 week, bacteria showing hydrolytic activity (a hole in the jellified medium for agarase or κ-carrageenase activity or complete liquefaction of the medium for ι-carrageenase or alginate lyase activity) were recovered and plated in order to obtain isolated colonies. Activity was confirmed by testing again the isolated colonies for the observed activity/activities.

### 16s rRNA sequence of the bacterial candidates showing polysaccharidase activity

**Figure 2:**
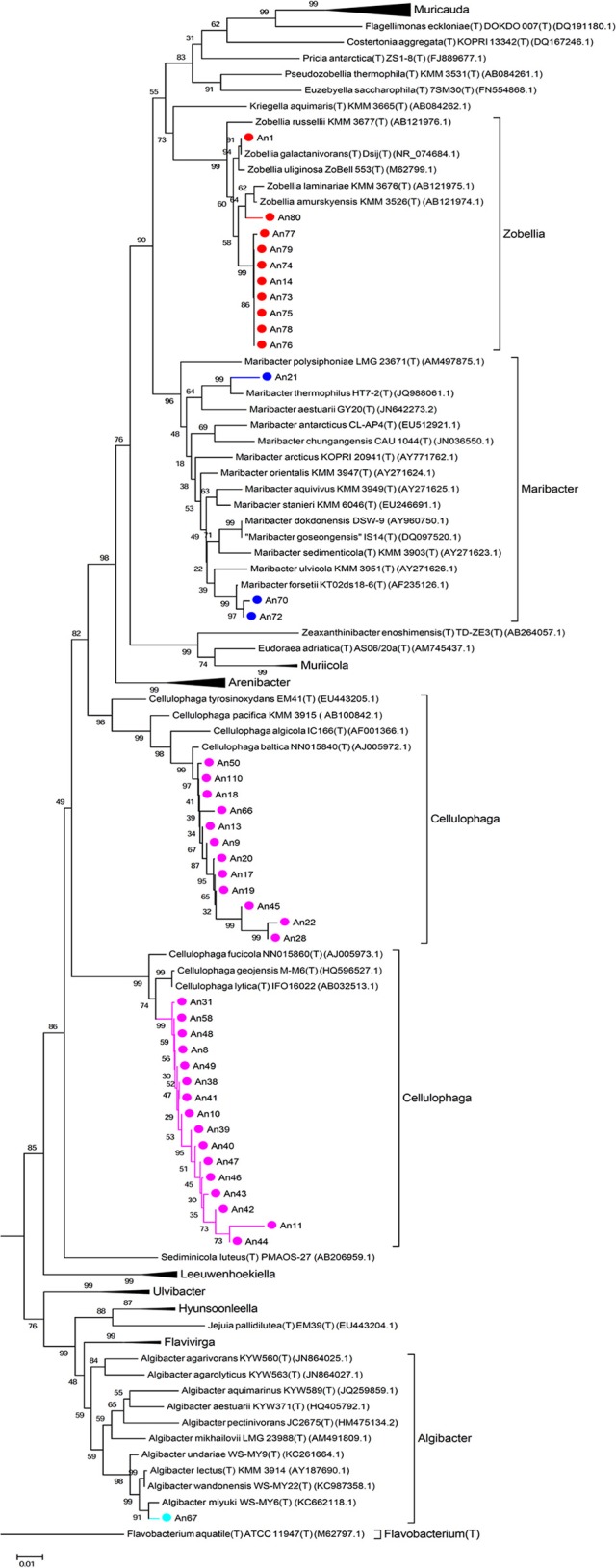
**Phylogenetic tree with the ***Flavobacteriaceae*****. 16S rRNA sequences of 120 representatives of the family *Flavobacteriaceae* were aligned with those of our 41 MAPD flavobacterial isolates. Sequences were manually curated and phylogenetic trees were constructed with these alignments using the Neighbor Joining Method. Evolutionary distances were calculated according to Kimura's two-parameter model using the gamma distribution (Kimura, 1980).

**Figure 3:**
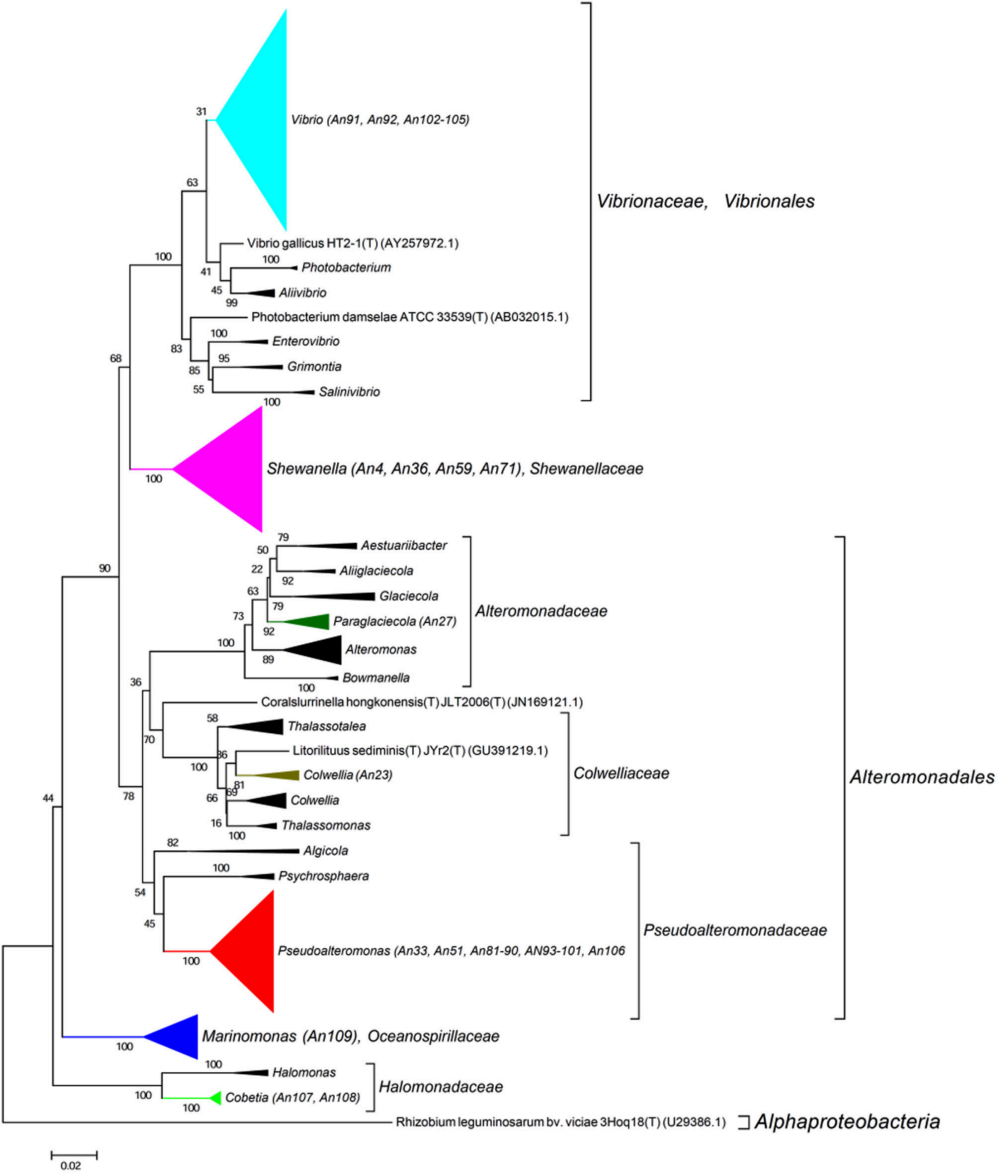
**Phylogenetic tree with the ***Gammaproteobacteria*****. 16S rRNA sequence of 355 representatives of the class *Gammaproteobacteria* were aligned with those of our 37 MAPD Gammaproteobacterial isolates. Sequence were manually curated and phylogenetic trees were constructed with these alignments using the Neighbor Joining Method. Evolutionary distances were calculated according to Kimura's two-parameter model using the gamma distribution (Kimura, 1980).

The 16S rRNA genes were amplified by PCR (Taq DNA polymerase, Roche) directly from the isolated colonies. Universal primers were used (8F, 5′-AGAGTTTGATCCTGGCTCAG-3′ and 1492R, 3′-TACGGCTACCTTGTTACGACTT-5′). The amplified genes were sequenced by Sanger sequencing from both ends (forward and reverse; GATC Biotech, Germany). A consensus sequence was produced from the overlapping reads, for the 16S rRNA gene of each MAPD isolate. For each sequence, low quality 5 and 3′ extremities were manually trimmed, in order to keep only high quality sequences (DNA base peaks with quality scores above 30). Finally, the BLAST program (NCBI) was used to compare the obtained sequences against the GenBank database and closest neighbors were found by alignment with type strains with verified species names using EzTaxon (Kim et al., 2012). Sequences were manually aligned and curated with a representative set of 120 sequences of identified members of the family *Flavobacteriaceae* (Figure 2) and 355 of the class *Gammaproteobacteria* (Figure 3, Figure S1). Phylogenetic trees were constructed with these alignments using the Neighbor Joining Method (Saitou and Nei, 1987). Evolutionary distances were calculated according to Kimura's two-parameter model using the gamma distribution (Kimura, 1980).

### Nucleotide sequence accession numbers

Sequence data of the V3-V4 region of the 16S rRNA gene of the non-MAPD isolates and the partial 16S rRNA of the 78 MAPD isolates were submitted to the EMBL database under accession numbers LN881131 to LN881422.

## Results and discussion

### The cultivable microbiota of *Ascophyllum nodosum*: comparison with the total bacterial community of brown algae and highlight of a host-specific common core

*A. nodosum* is a large brown alga (up to 2 m) of the *Fucaceae* family which is common on both sides of North Atlantic ocean (Olsen et al., 2010). This macroalga is dominant along sheltered intertidal rocky shores and is a very long-lived species (10–15 years). The microbial population of *A. nodosum* was studied long ago by culturing (Chan and McManus, 1969) and electronic microscopy (Cundell et al., 1977). Surprisingly, these two old studies are the only ones concerning microorganisms associated with this brown seaweed. Cundell et al. (1977) describe the microbial epiphytes of *A. nodosum* as “*a complex assemblage of end-attached bacteria, filamentous bacteria, flexibacteria, yeasts, and pennate diatoms*.” We here report the first extensive characterization of the cultivable bacterial community of this ecologically relevant macroalga.

Approximately 3.10^3^ bacteria were isolated from the surface of three *A. nodosum* thallus samples. A total of 324 bacteria were recovered (108/sample) for further analysis. We succeeded in amplifying and sequencing the V3-V4 16S rRNA region from a total of 297 bacteria (98 from S1, 97 from S2, 102 from S3). These sequences were aligned to those from the GenBank and SILVA SSU databases (data not shown), with 291 out of 297 isolates being assigned to a genera. As a proof of quality sequence alignments, we had high average query coverage of 97.2% and identity percentage of 98.9% for the V3-V4 region sequences of these isolates (Table S1). For each *A. nodosum* sample, tables were made with the relative abundance and the frequency of each bacterial genus, family, order, class, and phylum identified (Table S2).

In the total isolated bacterial population, 36 different genera were identified. The most abundantly represented were *Marinomonas* (13.1%), *Cellulophaga* (10.1%), and *Pseudoalteromonas* (9.1%). Eighteen bacterial families were found, the major ones being *Flavobacteriaceae* (42.8%), *Oceanospirillaceae* (15.5%), and *Rhodobacteraceae* (11.1%). The most abundant phylum was *Proteobacteria* (56%, with class *Alpha*: 11.4%, *Gamma:* 44.1%, *Beta*: 0.7%), closely followed by *Bacteroidetes* (class *Flavobacteriia* 42.8%). *Firmicutes* (1.0%) members were identified only on sample S3 and constituted a very minor fraction of the cultivable community.

Previous molecular studies on the kelp *Laminaria hyperborean* (Bengtsson and Øvreås, 2010; Bengtsson et al., 2012) and on *Fucus vesiculosis* (Lachnit et al., 2011; Stratil et al., 2013, 2014), closely related to *A. nodosum*, reveal the phyla- and class-level structures of their associated microbial communities to be markedly different from the here identified population (see for review Martin et al., 2014b). *Alphaproteobacteria* emerged as a dominant phylum of the total bacterial community of these molecular studies (25–60%), whereas it represents only a minor fraction of the isolated cultivable fraction (11%). In contrast, the abundance of *Bacteroidetes* and *Gammaproteobacteria* members is two- to three-fold higher in our cultivable fraction. Surprisingly, no *Planctomycetes* and *Cyanobacteria* were isolated in our cultivable population even if they represent dominant phyla in the above mentioned reports of microbiota associated with other brown algae. Concerning the absence of *Cyanobacteria*, to our knowledge no representative of this autotrophic class has yet been identified by culturing methods dealing with algal microbiotas (Hollants et al., 2013). Furthermore, as our samples were taken in March 2014, a seasonal shift is likely to explain both the absence of *Cyanobacteria* and the high percentage of *Gammaproteobacteria* members among our isolates, as previously observed for *F. vesiculosus* (Lachnit et al., 2011). The absence of isolated *Planctomycetes* is more surprising, as these heterotrophic bacteria are expected to grow readily on complex culture media.

At lower taxonomic rank, molecular studies have provided evidence that total bacterial communities can differ markedly from one individual alga to another and that the core community specific to the host is limited (~15%; Burke et al., 2011; Bengtsson et al., 2012). Here, looking solely at the cultivable community of *A. nodosum*, we reach partly similar conclusions as there is some variability between the communities isolated on the three samples, as regards both the genera observed and their relative abundances (Figure 1, Table S2). On the PCoA plot (Figure 1), one can see that the samples are equally distant (dissimilar in their bacterial composition) from each other (Bray-Curtis distances between S1–S2 = 0.45; S1–S3 = 0.412; S2–S3 = 0.453). Moreover, some genera are specific to one sample (e.g., *Hydrogenophaga, Ruegeria*, and *Tenacibaculum* were found only on S1) or common to just two samples (e. g., *Leucothrix, Psychroserpens*, and *Psychrobacter* were identified only on S2 and S3). However, despite this genus diversity, 12 genera were consistently found on all three *A. nosodum* samples: (i) six *Flavobacteriia* genera, all of the *Flavobacteriaceae* family (*Algibacter, Cellulophaga, Dokdonia, Formosa, Maribacter*, and *Zobellia*); (ii) five *Gammaproteobacteria* genera; four genera of the order *Alteromonadales* but of different families (*Colwellia, Glaciecola, Pseudoalteromonas*, and *Shewanella*) and one genus of the order *Oceanospirillales* (*Marinomonas, Oceanospirillaceae* family); and (iii) one *Alphaproteobacteria* genus (*Sulfitobacter, Rhodobacteraceae* family; Figure 1). These genera represent a core community of 33% of the cultivable subpopulation (12 out of 36 genera in total), which is twice the proportion observed in whole seaweed-associated microbiotas (Burke et al., 2011; Bengtsson et al., 2012). This common core includes several host-specific genera which are well known to associate with macroalgae. *Cellulophaga, Pseudolateromonas, Shewanella, Sulfitobacter*, and *Zobellia*, for example, are among the 33 genera isolated from all three macroalgal groups (brown, green, and red seaweeds), as determined on the basis of a review of 161 studies (mainly culture-based) dealing with bacteria associated with 159 macroalgal species (Hollants et al., 2013). *Formosa, Dokdonia, Marinomonas*, and *Glaciecola* have been also identified on other brown algae like *Fucus vesiculosus* (Lachnit et al., 2011; Goecke et al., 2013), *F. evanescens* (Ivanova et al., 2004a) or *Laminaria* sp. (Wiese et al., 2009; Dong et al., 2012). To our knowledge, *Colwellia* is the only genus retrieved here from our three alga samples (representing 3.4% of our total bacterial isolates) that has not previously been isolated from brown algae. The *Colwelliaceae* family (comprising the genera *Colwellia* and *Thalassomonas*) has already been identified on the red alga *Delisea pulchra* (Fernandes et al., 2012). Fernandes et al. found this family to be present on diseased thalli and absent from healthy thalli of this red alga.

### The cultivable microbiota from *A. nodosum* is enriched in MAPD bacteria: novel activities and putative novel species

The 324 isolated bacteria were plated and tested on culture media gelified with agar, κ-carrageenan, ι-carrageenan, or alginate salts. We have found 78 isolates (24%) with polysaccharidase activity against at least one of the tested substrates (Table 1). Similar proportions of such bacteria were isolated from the three specimens collected (S1, 20%; S2, 23%; S3, 34%). Thus our study clearly indicates that the cultivable microbiota of *A. nodosum* is significantly enriched in MAPD bacteria as compared to the total bacterial community (based on molecular studies and on to the difficulty to identify MAPD enzymes by functional genomics). Nonetheless, MAPD bacteria remain a minority even within the cultivable microbiota (24%).

**Table 1:** **Identified MAPD isolates, each with the closest type strain, the corresponding percentage of 16S rRNA gene similarity and the observed polysaccharidase activities**.

**Isolated bacterial strain**	**Closest bacterial strain (EZTaxon)**	**% identity**	**Polysaccharidase activities^a^**				
			**Ag**		**í-C**	**κ-C**	**AL**
***FLAVOBACTERIIA***							
***FLAVOBACTERIACEAE***							
***ALGIBACTER* sp**.							
An67	*Algibacter miyuki* WS-MY6	97.75	**+**		**+**	**–**	**–**
***CELLULOPHAGA*** **sp**.							
An8	*Cellulophaga geojensis* M-M6	97.88	**+**		**+**	**+**	**+**
An10	*Cellulophaga geojensis* M-M6	97.88	**+**		**+**	**+**	**+**
An42	*Cellulophaga geojensis* M-M6	97.66	**+**		**+**	**+**	**–**
An44	*Cellulophaga geojensis* M-M6	96.28	**+**		**+**	**+**	**+**
An47	*Cellulophaga geojensis* M-M6	96.6	**+**		**+**	**+**	**–**
An48	*Cellulophaga geojensis* M-M6	97.65	**+**		**+**	**+**	**–**
An11	*Cellulophaga fucicola* NN015860	95.56	**+**		**+**	**+**	**+**
An31	*Cellulophaga fucicola* NN015860	97.22	**+**		**+**	**+**	**+**
An38	*Cellulophaga fucicola* NN015860	97.51	**+**		**+**	**+**	**+**
An39	*Cellulophaga fucicola* NN015860	98	**+**		**+**	**+**	**+**
An40	*Cellulophaga fucicola* NN015860	96.75	**+**		**+**	**+**	**+**
An41	*Cellulophaga fucicola* NN015860	97.58	**+**		**+**	**+**	**+**
An43	*Cellulophaga fucicola* NN015860	96.83	**+**		**+**	**+**	**+**
An46	*Cellulophaga fucicola* NN015860	96.82	**+**		**+**	**+**	**–**
An49	*Cellulophaga fucicola* NN015860	97.65	**+**		**+**	**+**	**+**
An9	*Cellulophaga baltica* NN015840	98.27	**+**		**+**	**+**	**+**
An13	*Cellulophaga baltica* NN015840	98.64	**+**		**+**	**+**	**+**
An17	*Cellulophaga baltica* NN015840	97.75	**+**		**+**	**+**	**+**
An18	*Cellulophaga baltica* NN015840	98.49	**+**		**+**	**+**	**+**
An19	*Cellulophaga baltica* NN015840	98.20	**+**		**+**	**+**	**+**
An20	*Cellulophaga baltica* NN015840	97.45	**+**		**+**	**+**	**+**
An22	*Cellulophaga baltica* NN015840	95.87	**+**		**+**	**+**	**–**
An28	*Cellulophaga baltica* NN015840	96.32	**+**		**+**	**+**	**+**
An45	*Cellulophaga baltica* NN015840	98.63	**+**		**+**	**+**	**+**
An50	*Cellulophaga baltica* NN015840	97.68	**+**		**+**	**+**	**+**
An110	*Cellulophaga baltica* NN015840	98.53	**+**		**+**	**+**	**+**
An66	*Cellulophaga pacifica* KMM3664	96.21	**+**		**+**	**+**	**+**
***MARIBACTER*** **sp**.							
An21	*Maribacter aestuarii* GY20	95.33	**+**		**+**	**+**	**+**
An70	*Maribacter forsetii* KT02ds 18-6	99.28	**–**		**+**	**–**	**+**
An72	*Maribacter forsetii* KT02ds 18-6	99.06	**–**		**+**	**–**	**+**
***ZOBELLIA*** **sp**.							
An1	*Zobellia galactanivorans* DsiJ	99.4	**+**		**+**	**+**	**+**
An14	*Zobellia galactanivorans* DsiJ	97.63	**+**		**+**	**–**	**–**
An73	*Zobellia galactanivorans* DsiJ	97.7	**+**		**+**	**–**	**+**
An75	*Zobellia galactanivorans* DsiJ	98	**+**		**+**	**–**	**–**
An76	*Zobellia galactanivorans* DsiJ	97.8	**+**		**+**	**–**	**+**
An78	*Zobellia galactanivorans* DsiJ	97.5	**+**		**+**	**–**	**–**
An79	*Zobellia galactanivorans* DsiJ	97.8	**+**		**+**	**–**	**–**
An74	*Zobellia laminariae* KMM3676	97.6	**+**		**+**	**–**	**–**
An77	*Zobellia laminariae* KMM3676	96.8	**+**		**+**	**–**	**–**
An80	*Zobellia laminariae* KMM3676	98.9	**+**		**+**	**+**	**–**
***GAMMAPROTEOBACTERIA***							
***ALTEROMONADACEAE***							
***PARAGLACIECOLA*** **sp**.							
An27	*Paraglaciecola mesophila* KMM241	96.49		**+**	**+**	**+**	**–**
***COLWELLIACEAE***							
***COLWELLIA*** **sp**.							
An23	*Colwellia meonggei* MA1-3	96.17		**+**	**+**	**+**	**–**
***HALOMONODACEAE***							
***COBETIA*** **sp**.							
An107	*Cobetia litoralis* KMM 3880	99.31		**–**	**–**	**–**	**+**
An108	*Cobetia litoralis* KMM 3880	98.96		**–**	**–**	**–**	**+**
***OCEANOSPIRILLACEAE***							
***MARINOMONAS*** **sp**.							
An109	*Marinomonas ushuaiensis* U1	97.14		**–**	**–**	**–**	**+**
***PSEUDOALTEROMONODACEAE***							
***PSEUDOALTEROMONAS*** **sp**.							
An88	*Pseudoalteromonas aliena* KMM 3562	99.57		**–**	**–**	**–**	**+**
An89	*Pseudoalteromonas aliena* KMM 3562	99.2		**–**	**–**	**–**	**+**
An97	*Pseudoalteromonas aliena* KMM 3562	98.3		**–**	**–**	**–**	**+**
An33	*Pseudoalteromonas espejiana* NCIMB 2127	99.4		**–**	**+**	**+**	**+**
An51	*Pseudoalteromonas espejiana* NCIMB 2127	98.1		**–**	**–**	**–**	**+**
An81	*Pseudoalteromonas espejiana* NCIMB 2127	99.34		**–**	**–**	**–**	**+**
An82	*Pseudoalteromonas espejiana* NCIMB 2127	99.42		**–**	**–**	**–**	**+**
An83	*Pseudoalteromonas espejiana* NCIMB 2127	99.13		**–**	**–**	**–**	**+**
An84	*Pseudoalteromonas espejiana* NCIMB 2127	99.71		**–**	**+**	**+**	**+**
An85	*Pseudoalteromonas espejiana* NCIMB 2127	98.84		**–**	**–**	**–**	**+**
An86	*Pseudoalteromonas espejiana* NCIMB 2127	99.05		**–**	**–**	**–**	**+**
An87	*Pseudoalteromonas espejiana* NCIMB 2127	98.11		**–**	**–**	**–**	**+**
An90	*Pseudoalteromonas espejiana* NCIMB 2127	98.98		**–**	**+**	**–**	**+**
An93	*Pseudoalteromonas espejiana* NCIMB 2127	99.6		**–**	**–**	**–**	**+**
An94	*Pseudoalteromonas espejiana* NCIMB 2127	98.8		**–**	**–**	**–**	**+**
An95	*Pseudoalteromonas espejiana* NCIMB 2127	99.7		**–**	**–**	**–**	**+**
An99	*Pseudoalteromonas espejiana* NCIMB 2127	99.7		**–**	**+**	**+**	**+**
An100	*Pseudoalteromonas espejiana* NCIMB 2127	99.6		**–**	**+**	**+**	**+**
An101	*Pseudoalteromonas espejiana* NCIMB 2127	98.1		**–**	**–**	**–**	**+**
An106	*Pseudoalteromonas espejiana* NCIMB 2127	98.76		**–**	**–**	**–**	**+**
An96	*Pseudolateromonas nigrifaciens* NCIMB 8614	99.2		**–**	**–**	**–**	**+**
An98	*Pseudolateromonas nigrifaciens* NCIMB 8615	99.2		**–**	**–**	**–**	**+**
***SHEWANELLACEAE***							
***SHEWANELLA*** **sp**.							
An36	*Shewanella pacifica* KMM3597	98.3		**–**	**+**	**+**	**+**
An59	*Shewanella pacifica* KMM3597	97.6		**–**	**+**	**+**	**+**
An71	*Shewanella pacifica* KMM3597	97.6		**–**	**+**	**+**	**+**
An4	*Shewanella japonica* KMM 3299	98.3		**–**	**+**	**–**	**+**
***VIBRIONACEAE***							
***VIBRIO*** **sp**.							
An91	*Vibrio hemicentroti* AlyHp32	99.17		**–**	**–**	**–**	**+**
An92	*Vibrio splendidus* ATCC 33125	97.2		**–**	**–**	**–**	**+**
An102	*Vibrio splendidus* ATCC 33125	98.99		**–**	**–**	**–**	**+**
An103	*Vibrio splendidus* ATCC 33125	99.14		**–**	**–**	**–**	**+**
An104	*Vibrio splendidus* ATCC 33125	99.21		**–**	**–**	**–**	**+**
An105	*Vibrio splendidus* ATCC 33125	99.28		**–**	**–**	**–**	**+**

*16S rRNA perecentage lower than 97% were indicated in red*.

a *Ag, agarase; í-C, ι-carrageenase; κ-C, κ-carrageenase; AL, alginate lyase*.

The entire 16S rRNA genes of the various candidates were amplified and sequenced. Sequences were aligned with those of type strains with valid species names (EzTaxon, Kim et al., 2012). The percentage of identity to the closest type strain and the polysaccharidase activities of each isolate are listed in Table 1. The first remarkable result is that all the MAPD isolates belong to only two bacterial classes, despite the taxonomic diversity of the cultivable community: 41 isolates were identified as *Flavobacteriia* members and 37 as *Gammaproteobacteria* members (Table 1).

**Figure 4:**
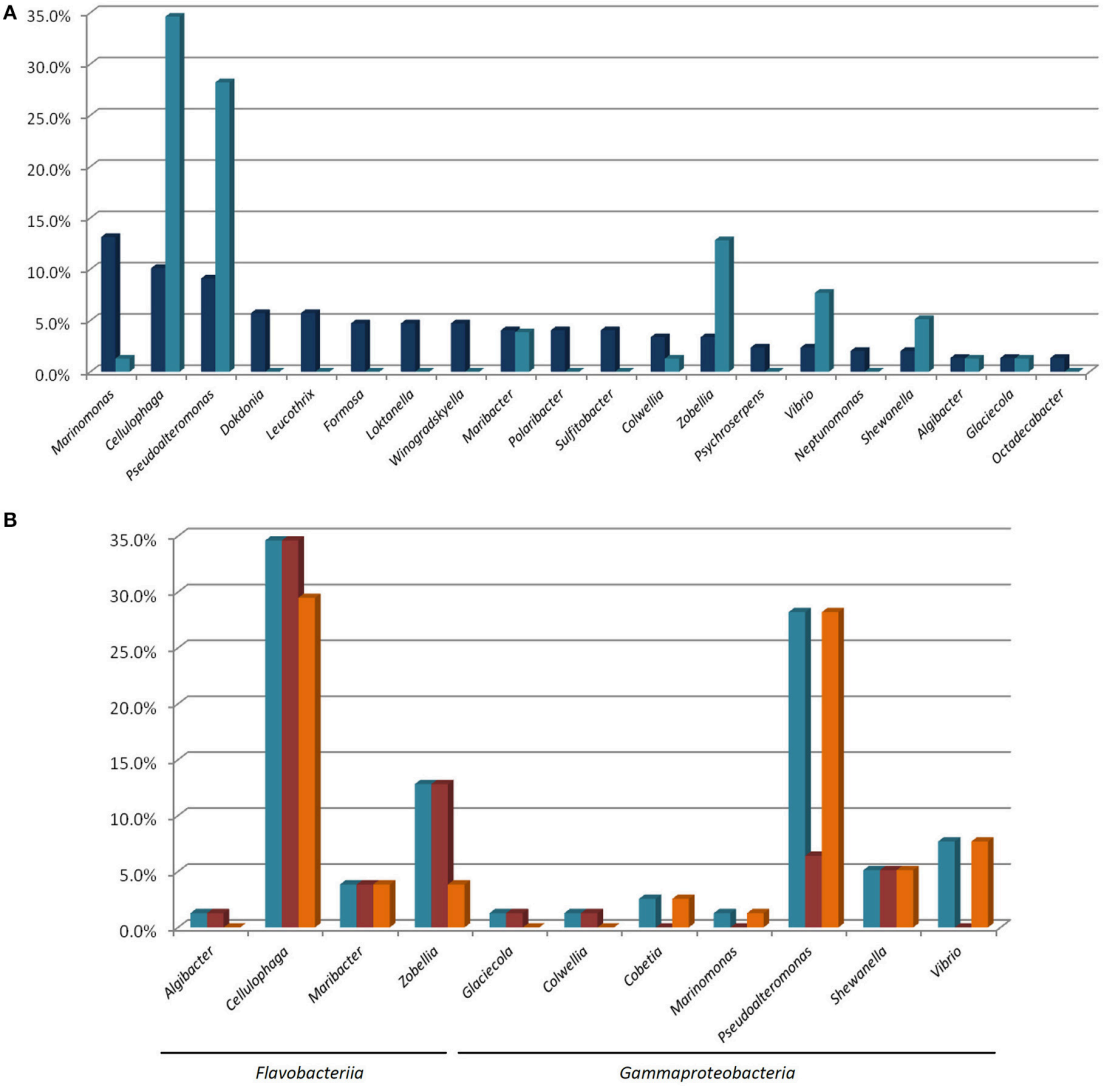
**(A)** Percentage proportions of the most represented genera in the total isolated bacterial population (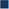) and of the MAPD isolates belonging to these genera in the whole set of 78 MAPD isolates (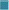); **(B)** Percentage proportions of MAPD isolates belonging to each MAPD-isolate-containing genus in the whole set of 78 MAPD isolates (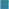), with their activities on red (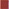) or brown seaweed galactans (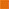).

These isolates were assigned to 11 genera (out of the 36 genera isolated) and 8 families. Among the ten most abundant genera in our total isolated population (Figure 4A), only four (*Marinomonas, Cellulophaga, Pseudoalteromnas*, and *Maribacter*) are represented by isolates with at least one polysaccharolytic activity. The genera *Cellulophaga* and *Pseudoalteromonas* are exceptional, being both abundant in the cultivable community (the second and third most abundant genera, respectively), and representing together more than 60% of the MAPD isolates (27/78 and 22/78, respectively; Figures 4A,B). In contrast, the most abundant genus (*Marinomonas*) is represented here by only one MAPD isolate (with a single, alginolytic activity). Similarly, *Formosa* is the 6th most abundant genus, but none of the *Formosa* isolates displays any MAPD activity. This is particularly unexpected, since the characterized species *Formosa agariphila* is agarolytic (Nedashkovskaya, 2006) and its genome analysis has revealed a broad potential for degrading algal polysaccharides (Mann et al., 2013). This striking example is a reminder that extrapolating the biological or ecological function of a particular species to a larger bacterial group can lead to incorrect interpretations. Among the less abundant genera, *Zobellia* (13th, 10/297), *Vibrio* (15th, 7/297), and *Shewanella* (17th, 6/297) are also remarkable by their overrepresentation among the MAPD isolates (Figure 4B). Notably, 100% of the *Zobellia* isolates display MAPD activity.

Interestingly, the polysaccharidase activities identified here do not necessarily reflect the cell wall composition of *A. nodosum*. Brown alga cell walls are largely composed of alginates and sulfated fucoidans, whereas red macroalgae mainly contain sulfated galactans (agars or carrageenans; Popper et al., 2011). Unexpectedly, we have found similar proportions of bacterial isolates degrading polysaccharides of red (64/78) and brown (61/78) macroalgae (Figure 4B, Table 1), but these activities are not equally distributed between *Gammaproteobacteria* and *Flavobacteriia*. Most gammaproteobacterial isolates (26/37) exclusively degrade alginate (including all *Cobetia, Marinomonas*, and *Vibrio* isolates). All *Pseudoalteromonas* isolates are alginolytic, but a minority is also able to hydrolyze carrageenans (22%), and none of them is agarolytic. MAPD activities are more evenly distributed in *Shewanella*, all isolates being both alginolytic and carrageenolytic. More surprisingly, the *Paraglaciecola* and *Colwellia* isolates are not alginolytic, but can hydrolyze both agars and carrageenans. In contrast, the *Flavobacteriia* isolates are much more generalistic degraders. All the MAPD *Flavobacteriia* isolates hydrolyze red algal sulfated galactans (agars and/or carrageenans) and most of them (26/41) also alginate. This suggests that the MAPD *Gammaproteobacteria* isolates are more specific to brown algae, while the MAPD *Flavobacteriia* strains or species isolated from *A. nodosum* are likely to be found also on the surfaces of agarophytic and carrageenophytic red seaweeds.

**Figure 5:**
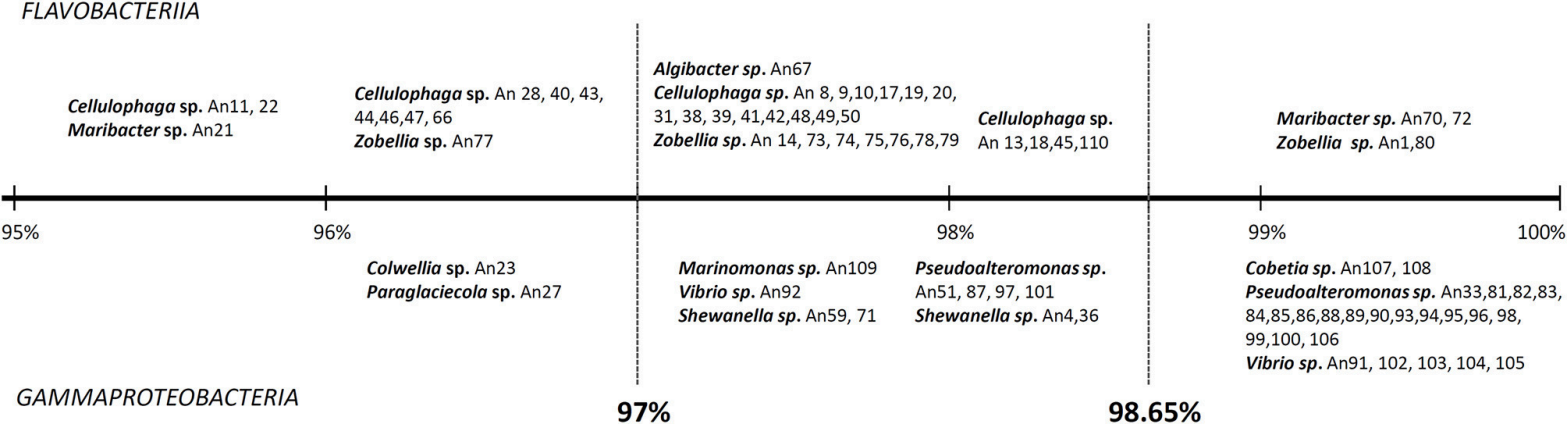
**Ranges of 16S rRNA identity percentages for the identified MAPD isolates vs. known species**. Two third of the MAPD isolates (< 98.65% 16S rRNA identities) likely represent novel species. Indeed, 97% is the commonly accepted threshold percentage at which two species can be distinguished and 98.65% is the threshold proposed by Kim et al. (2014) which have compared the average nucleotide identities of almost 7000 prokaryotic genomes and their 16S rRNA gene identities.

It is also noteworthy that we have discovered MAPD activities in genera that were not previously known to include MAPD bacteria (*Marinomonas* and *Colwellia* strains) or to display the activities observed here (ι- and κ-carrageeenases in *Algibacter* and *Maribacter* isolates, respectively; Table 2). These novel activities may be explained by the likely isolation of novel MAPD species. Indeed, 63% of the MAPD isolates identified here have less than 98.65% sequence identity at 16S rRNA level to a known species (Figure 5). Therefore they could represent putative novel species even if there is still some discussion regarding the threshold percentage of 16S rRNA gene identity at which two species can be distinguished. A commonly accepted value is 97%. Recently, Kim et al. (2014), having compared the average nucleotide identities of almost 7000 prokaryotic genomes and their 16S rRNA gene identities, propose a threshold of 98.65%, while Tindall et al. (2010) stress that the 16S rRNA alone does not describe a species but only provides a putative indication of a novel species. Nevertheless, further taxonomic analyses and DNA-DNA hybridization experiments should be performed or average nucleotide identities determined to confirm this (Tindall et al., 2010; Stackebrandt, 2011). However, to strengthen the taxonomic identification of these MAPD isolates and the assumption that most of these isolates represent new species, phylogenetic trees of entire 16S rRNA genes were constructed for the *Flavobactericeae* (Figure 2) and *Gammaproteobacteria* members (Figure 3, Figure S1). The *Flavobacteriaceae* phylogenetic tree strongly suggests that we have identified three novel *Zobellia* species (represented by An80, An77, and the seven strains of the An14 clade) and a novel *Maribacter* species (An21; Figure 2). Furthermore, in this phylogenetic tree, the *Cellulophaga* genus clearly appears non-monophyletic as the MAPD *Cellulophaga* isolates are separated into two clades, 12 of them having a common ancestor with *C. baltica* and the other 16 a common ancestor with *C. lytica*. This result appears to confirm the doubts raised in Bergey's manual of Systematic Bacteriology concerning the monophyletic character of the *Cellulopha* genus (Krieg et al., 2011). In the detailed *Gammaproteobacteria* tree (Figure S1), the *Colwellia* sp. An23, the *Paraglaciecola* sp. An27, the four *Shewanella* isolated and the *Marinomonas* sp. An 109 seem very likely to represent novel species.

**Table 2:** **Activities identified in our study and previously described for MAPD species or strains from the genera to which our 78 MAPD isolates were assigned**.

**Genera to which the 78 MAPD-isolates were assigned**	**Activities identified in our study^a^**				**Previously described activities^a^**				**References**
	**Ag**	**í-C**	**κ-C**	**AL**	**Ag**	**í-C**	**κ-C**	**AL**	
***FLAVOBACTERIIA***									
*Algibacter*	•	■			•			•	Park et al., 2013; Tanaka et al., 2015
*Cellulophaga*	•	•	•	•	•	•	•	•	Johansen et al., 1999; Park et al., 2012; Yao et al., 2013
*Maribacter*	•	•	■	•	•	•		•	Barbeyron et al., 2008
*Zobellia*	•	•	•	•	•	•	•	•	Barbeyron et al., 2001; Nedashkovskaya et al., 2004
***GAMMAPROTEOBACTERIA***									
*Paraglaciecola*	•	•	•		•	•	•		Romanenko et al., 2003; Yong et al., 2007
*Colwellia*	■	■	■						Browman, 2013; Liu et al., 2014; Wang et al., 2015
*Cobetia*				•				•	Lelchat et al., 2015
*Marinomonas*				■					Macián et al., 2005; Lucas-Elió et al., 2011
*Pseudolateromonas*		•	•	•	•	•	•	•	Akagawa-Matsushita et al., 1992; Chi et al., 2014
*Shewanella*		•	•	•	•	•	•	•	Ivanova et al., 2001, 2003, 2004b; Wang et al., 2014
*Vibrio*				•	•			•	Sugano et al., 1993; Kim et al., 2013

• *Activities found in our study and found previously for species/strains of this genus*;

■ *Novel activities, that weren't identified for any species/strains of this genus previously*.

a *Ag, agarase; í-C, ι-carrageenase; κ-C, κ-carrageenase; AL, alginate lyase*.

Last but not least, one can observe that the proportion of MAPD bacteria increases dramatically while looking only at the core group of cultivable bacteria. Indeed, MAPD activity was detected in 75% of the core genera (*Algibacter, Cellulophaga, Colwellia, Glaciecola Maribacter, Marinomonas, Pseudoalteromonas, Shewanella* and *Zobellia*; Figure 1, Table 1). Thus, even though MAPD bacteria constitute a minor fraction of both the total and cultivable bacterial communities, they apparently belong to the core group of bacteria living at the surface of *A. nodosum* and likely exert functions that are important for their macroalgal host and/or within the microbiota as a whole. How harboring MAPD bacteria might be beneficial to the host is not obvious. Such bacteria have mostly been described as detrimental to macroalgae, being responsible for diseases, providing an entry for opportunistic bacteria, or accelerating algal degradation (Goecke et al., 2010; Egan et al., 2013; Hollants et al., 2013). Recently, Marzinelli et al. (2015) compared microbial communities on healthy and bleached thalli of the brown kelp *Ecklonia radiata*. They found *Flavobacteriaceae* and *Oceanospirillaceae* representatives to be more present on diseased tissues. Within these families, however, some genera were found in much higher proportion on healthy samples than on bleached ones, suggesting a role favorable to the macroalgal host. Interestingly, these genera include several of those represented by MAPD isolates obtained from *A. nodosum*: *Zobellia, Maribacter, Pseudoalteromonas, Vibrio, Marinomonas*, and *Cobetia*. Beyond their MAPD activities, species of these genera may have additional metabolic capacities advantageous for their hosts. This hypothesis is plausible at least for *Zobellia* species, which are known to synthesize an algal morphogenesis inducer (Matsuo et al., 2003). The role of MAPD bacteria within the total seaweed-associated microbiota is more obvious. These bacteria are essential for degrading intact cell-wall polysaccharides, and thus for releasing hydrolysis products assimilable by the much more abundant bacteria (e.g., *Alphaproteobacteria*) lacking these unique MAPD enzymes.

## Conclusion

In terrestrial environments, the bacteria involved in recycling plant polysaccharides are essentially found both on living plants and in the soils that immediately surround them. The situation is more complex for marine macroalgae. They live attached to rocks, and when algal fragments are released, they are quickly dispersed by the waves and tides. The available marine metagenomic data show that the water column is a habitat poor in MAPD bacteria and, for a macroalga, not equivalent to a surrounding soil. Tidal sediments could be crucial reservoirs of MAPD bacteria, but this remains an open question. A third environment likely to be a habitat for MAPD bacteria is the surface of the macroalgae themselves. We have shown here that this is indeed the case and that the cultivable microbiota of healthy *A. nodosum* specimens is enriched in MAPD bacteria. These bacteria, however, are not the most abundant ones associated with brown seaweeds; they constitute a minority fraction even within the cultivable subpopulation. An attractive hypothesis is that this low abundance of MAPD bacteria is due to active and/or passive defense systems of the macroalga, preventing proliferation of these potentially harmful bacteria. Evidence of such defense systems in macroalgae has been accumulating over the last decade (Potin et al., 2002; Egan et al., 2014). If this hypothesis is correct, one can expect MAPD bacteria to bloom on weakened or dead macroalgae, thus contributing significantly to recycling of macroalgal biomass. As regards bioprospecting, our work demonstrates that culturing (combined, for instance, with subsequent genome sequencing of cultivable isolates) is an efficient strategy for finding new MAPD bacteria and their corresponding polysaccharidases.

## Author contributions

Conceived and designed the experiments: MM, MV, GM, TB, DP. Performed the experiments: MM, RM. Analyzed the data: MM, MV, GM, TB. Contributed reagents/materials/analysis tools: MM, RM. Wrote the manuscript: MM, GM.

## Funding

This project was funded by Gembloux Agro-Bio Tech (ULg), Wallonie-Bruxelles International (WBI), and the Fonds Scientifique de la Recherche (F.R.S-F.N.R.S) in the framework of the Collaboration Program Hubert Curien. GM and TB are grateful for support by the French Government through the National Research Agency with regard to the “Blue Enzymes” ANR project with reference ANR-14-CE19-0020-01.

### Conflict of interest statement

The authors declare that the research was conducted in the absence of any commercial or financial relationships that could be construed as a potential conflict of interest.

## Supplementary material

Table S1 **Percentage ranges and averages of the query coverages and identity percentages for the alignments with the 291 V3-V4 region sequences of the isolates to which a genus have been attributed**.

Table S2 **Abundance (#) and percentage proportion of each genus, family, order, class, and phylum identified on each sample**.

Figure S1 **Detailed phylogenetic tree for the ***Gammaproteobacteria*****.

## Abbreviations

MAPD: Macroalgal-polysaccharide-degrading bacteria.

## References

[B1] Akagawa-Matsushita M. Matsuo M. Koga Y. Yamasato K. (1992). Alteromonas atlantica sp. nov. and Alteromonas carrageenovora sp. nov., bacteria that decompose algal polysaccharides. Int. J. Syst. Bacteriol. 42, 621–627. 10.1099/00207713-42-4-621

[B2] Barbeyron T. Carpentier F. L'Haridon S. Schüler M. Michel G. Amann R. (2008). Description of maribacter forsetii sp. nov., a marine *Flavobacteriaceae* isolated from North Sea water, and emended description of the genus Maribacter. Int. J. Syst. Evol. Microbiol. 58, 790–797. 10.1099/ijs.0.65469-0 18398171

[B3] Barbeyron T. L'Haridon S. Corre E. Kloareg B. Potin P. (2001). Zobellia galactanovorans gen. nov., sp. nov., a marine species of *Flavobacteriaceae* isolated from a red alga, and classification of [Cytophaga] uliginosa (ZoBell and Upham 1944) Reichenbach 1989 as Zobellia uliginosa gen. nov., comb. nov. Int. J. Syst. Evol. Microbiol. 51, 985–997. 10.1099/00207713-51-3-985 11411725

[B4] Barbeyron T. Michel G. Potin P. Henrissat B. Kloareg B. (2000). Iota-Carrageenases constitute a novel family of glycoside hydrolases, unrelated to that of kappa-carrageenases. J. Biol. Chem. 275, 35499–35505. 10.1074/jbc.M003404200 10934194

[B5] Bengtsson M. M. Øvreås L. (2010). Planctomycetes dominate biofilms on surfaces of the kelp Laminaria hyperborea. BMC Microbiol. 10:261. 10.1186/1471-2180-10-261 20950420 PMC2964680

[B6] Bengtsson M. M. Sjøtun K. Lanzén A. Øvreås L. (2012). Bacterial diversity in relation to secondary production and succession on surfaces of the kelp Laminaria hyperborea. ISME J. 6, 2188–2198. 10.1038/ismej.2012.67 22763650 PMC3505018

[B7] Browman J. P. (2013). The family colwelliaceae, in The Prokaryotes: Gammaproteobacteria, eds Rosenberg E. DeLong E. F. Lory S. Stackebrandt E. Thompson F. (Berlin; Heidelberg: Springer), 179–195.

[B8] Bulgarelli D. Schlaeppi K. Spaepen S. Ver Loren van Themaat E. Schulze-Lefert P. (2013). Structure and functions of the bacterial microbiota of plants. Annu. Rev. Plant Biol. 64, 807–838. 10.1146/annurev-arplant-050312-120106 23373698

[B9] Burke C. Thomas T. Lewis M. Steinberg P. Kjelleberg S. (2011). Composition, uniqueness and variability of the epiphytic bacterial community of the green alga Ulva australis. ISME J. 5, 590–600. 10.1038/ismej.2010.164 21048801 PMC3105733

[B10] Chan E. C. McManus E. A. (1969). Distribution, characterization, and nutrition of marine microorganisms from the algae Polysiphonia lanosa and Ascophyllum nodosum. Can. J. Microbiol. 15, 409–420. 10.1139/m69-073 5786779

[B11] Chi W. J. Park J. S. Kang D. K. Hong S. K. (2014). Production and characterization of a novel thermostable extracellular agarase from Pseudoalteromonas hodoensis newly isolated from the west sea of South Korea. Appl. Biochem. Biotechnol. 173, 1703–1716. 10.1007/s12010-014-0958-3 24879592

[B12] Colin S. Deniaud E. Jam M. Descamps V. Chevolot Y. Kervarec N.. (2006). Cloning and biochemical characterization of the fucanase FcnA: definition of a novel glycoside hydrolase family specific for sulfated fucans. Glycobiology 16, 1021–1032. 10.1093/glycob/cwl029 16880504

[B13] Cundell A. M. Sleeter T. D. Mitchell R. (1977). Microbial populations associated with the surface of the brown alga ascophyllum nodosum. Microb. Ecol. 4, 81–91. 10.1007/BF02010431 24231887

[B14] DeAngelis K. M. Gladden J. M. Allgaier M. D'haeseleer P. Fortney J. L. Reddy A. (2010). Strategies for enhancing the effectiveness of metagenomic-based enzyme discovery in lignocellulolytic microbial communities. Bioenerg. Res. 3, 146–158. 10.1007/s12155-010-9089-z

[B15] de Oliveira L. Gregoracci G. Silva G. G. Salgado L. Filho G. Alves-Ferreira M.. (2012). Transcriptomic analysis of the red seaweed Laurencia dendroidea (Florideophyceae, Rhodophyta) and its microbiome. BMC Genomics 13:487. 10.1186/1471-2164-13-487 22985125 PMC3534612

[B16] Dixon P. (2003). VEGAN, a package of R functions for community ecology. J. Veg. Sci. 14, 927–930. 10.1111/j.1654-1103.2003.tb02228.x

[B17] Dong S. Yang J. Zhang X. Y. Shi M. Song X. Y. Chen X. L.. (2012). Cultivable alginate lyase-excreting bacteria associated with the arctic brown alga Laminaria. Mar. Drugs 10, 2481–2491. 10.3390/md10112481 23203272 PMC3509530

[B18] Egan S. Fernandes N. D. Kumar V. Gardiner M. Thomas T. (2014). Bacterial pathogens, virulence mechanism and host defence in marine macroalgae. Environ. Microbiol. 16, 925–938. 10.1111/1462-2920.12288 24112830

[B19] Egan S. Harder T. Burke C. Steinberg P. Kjelleberg S. Thomas T. (2013). The seaweed holobiont: understanding seaweed-bacteria interactions. FEMS Microbiol. Rev. 37, 462–476. 10.1111/1574-6976.12011 23157386

[B20] Fernandes N. Steinberg P. Rusch D. Kjelleberg S. Thomas T. (2012). Community structure and functional gene profile of bacteria on healthy and diseased thalli of the red seaweed Delisea pulchra. PLoS ONE 7:e50854. 10.1371/journal.pone.0050854 23226544 PMC3513314

[B21] Ferrer M. Martínez-Martínez M. Bargiela R. Streit W. R. Golyshina O. V. Golyshin P. N. (2015). Estimating the success of enzyme bioprospecting through metagenomics: current status and future trends. Microb. Biotechnol. [Epub ahead of print]. Available online at: http://onlinelibrary.wiley.com/doi/10.1111/1751-7915.12309/abstract 10.1111/1751-7915.12309 PMC4720405 26275154

[B22] Flament D. Barbeyron T. Jam M. Potin P. Czjzek M. Kloareg B.. (2007). Alpha-agarases define a new family of glycoside hydrolases, distinct from beta-agarase families. Appl. Environ. Microbiol. 73, 4691–4694. 10.1128/AEM.00496-07 17513582 PMC1932826

[B23] Gibson D. M. King B. C. Hayes M. L. Bergstrom G. C. (2011). Plant pathogens as a source of diverse enzymes for lignocellulose digestion. Curr. Opin. Microbiol. 14, 264–270. 10.1016/j.mib.2011.04.002 21536481

[B24] Goecke F. Labes A. Wiese J. Imhoff J. F. (2013). Phylogenetic analysis and antibiotic activity of bacteria isolated from the surface of two co-occurring macroalgae from the Baltic Sea. Eur. J. Phycol. 48, 47–60. 10.1080/09670262.2013.767944

[B25] Goecke F. Labes A. Wiese J. Imhoff J. (2010). Chemical interactions between marine macroalgae and bacteria. Mar. Ecol. Prog. Ser. 409, 267–299. 10.3354/meps08607

[B26] Gómez-Pereira P. R. Schüler M. Fuchs B. M. Bennke C. Teeling H. Waldmann J.. (2012). Genomic content of uncultured Bacteroidetes from contrasting oceanic provinces in the North Atlantic Ocean. Environ. Microbiol. 14, 52–66. 10.1111/j.1462-2920.2011.02555.x 21895912

[B27] Hehemann J.-H. Correc G. Barbeyron T. Helbert W. Czjzek M. Michel G. (2010). Transfer of carbohydrate-active enzymes from marine bacteria to Japanese gut microbiota. Nature 464, 908–912. 10.1038/nature08937 20376150

[B28] Hollants J. Leliaert F. De Clerck O. Willems A. (2013). What we can learn from sushi: a review on seaweed-bacterial associations. FEMS Microbiol. Ecol. 83, 1–16. 10.1111/j.1574-6941.2012.01446.x 22775757

[B29] Ivanova E. P. Alexeeva Y. V. Flavier S. Wright J. P. Zhukova N. V. Gorshkova N. M.. (2004a). Formosa algae gen. nov., sp. nov., a novel member of the family *Flavobacteriaceae*. Int. J. Syst. Evol. Microbiol. 54, 705–711. 10.1099/ijs.0.02763-0 15143012

[B30] Ivanova E. P. Gorshkova N. M. Bowman J. P. Lysenko A. M. Zhukova N. V. Sergeev A. F.. (2004b). Shewanella pacifica sp. nov., a polyunsaturated fatty acid-producing bacterium isolated from sea water. Int. J. Syst. Evol. Microbiol. 54, 1083–1087. 10.1099/ijs.0.02993-0 15280273

[B31] Ivanova E. P. Sawabe T. Gorshkova N. M. Svetashev V. I. Mikhailov V. V. Nicolau D. V.. (2001). Shewanella japonica sp. nov. Int. J. Syst. Evol. Microbiol. 51, 1027–1033. 10.1099/00207713-51-3-1027 11411670

[B32] Ivanova E. P. Sawabe T. Hayashi K. Gorshkova N. M. Zhukova N. V. Nedashkovskaya O. I.. (2003). Shewanella fidelis sp. nov., isolated from sediments and sea water. Int. J. Syst. Evol. Microbiol. 53, 577–582. 10.1099/ijs.0.02198-0 12710629

[B33] Johansen J. E. Nielsen P. Sjraholm C. (1999). Description of Cellulophaga baltica gen. nov., sp. nov. and Cellulophaga fucicola gen. nov.,sp. nov. and reclassification of [Cytophaga] Iytica to Cellulophaga lytica gen. nov., comb. nov. Int. J. Syst. Bacteriol. 49, 1231–1240. 10.1099/00207713-49-3-1231 10425785

[B34] Kim D. Baik K. S. Hwang Y. S. Choi J. S. Kwon J. Seong C. N. (2013). Vibrio hemicentroti sp. nov., an alginate lyase-producing bacterium, isolated from the gut microflora of sea urchin (Hemicentrotus pulcherrimus). Int. J. Syst. Evol. Microbiol. 63, 3697–3703. 10.1099/ijs.0.047951-0 23625262

[B35] Kim M. Oh H.-S. Park S.-C. Chun J. (2014). Towards a taxonomic coherence between average nucleotide identity and 16S rRNA gene sequence similarity for species demarcation of prokaryotes. Int. J. Syst. Evol. Microbiol. 64, 346–351. 10.1099/ijs.0.059774-0 24505072

[B36] Kim O. S. Cho Y. J. Lee K. Yoon S. H. Kim M. Na H.. (2012). Introducing EzTaxon-e: a prokaryotic 16s rRNA gene sequence database with phylotypes that represent uncultured species. Int. J. Syst. Evol. Microbiol. 62, 716–721. 10.1099/ijs.0.038075-0 22140171

[B37] Kimura M. (1980). A simple method for estimating evolutionary rates of base substitutions through comparative studies of nucleotide sequences. J. Mol. Evol. 16, 111–120. 10.1007/BF01731581 7463489

[B38] Krieg N. R. Staley J. T. Brown D. R. Hedlund B. P. Paster B. J. Ward N. L. (2011). Bergey's Manual® of Systematic Bacteriology: The Bacteroidetes, Spirochaetes, Tenericutes (Mollicutes), Acidobacteria, Fibrobacteres, Fusobacteria, Dictyoglomi, Gemmatimonadetes, Lentisphaerae, Verrucomicrobia, Chlamydiae, and Planctomycetes, 2nd Edn., Vol.4 New York, NY; Dordrecht; Heidelberg; London: Springer.

[B39] Lachnit T. Meske D. Wahl M. Harder T. Schmitz R. (2011). Epibacterial community patterns on marine macroalgae are host-specific but temporally variable. Environ. Microbiol. 13, 655–665. 10.1111/j.1462-2920.2010.02371.x 21078035

[B40] Lee H. S. Kwon K. K. Kang S. G. Cha S.-S. Kim S.-J. Lee J.-H. (2010). Approaches for novel enzyme discovery from marine environments. Curr. Opin. Biotechnol. 21, 353–357. 10.1016/j.copbio.2010.01.015 20189795

[B41] Lelchat F. Cérantola S. Brandily C. Colliec-Jouault S. Baudoux A.-C. Ojima T.. (2015). The marine bacteria Cobetia marina DSMZ 4741 synthesizes an unexpected K-antigen-like exopolysaccharide. Carbohydr. Polym. 124, 347–356. 10.1016/j.carbpol.2015.02.038 25839829

[B42] Leung H. T. C. Maas K. R. Wilhelm R. C. Mohn W. W. (2015). Long-term effects of timber harvesting on hemicellulolytic microbial populations in coniferous forest soils. ISME J. [Epub ahead of print]. Available online at: http://www.nature.com/ismej/journal/vaop/ncurrent/full/ismej2015118a.html 10.1038/ismej.2015.118 PMC4737928 26274049

[B43] Liu Y. Liu L.-Z. Zhong Z.-P. Zhou Y.-G. Liu Y. Liu Z.-P. (2014). Colwellia aquaemaris sp. nov., isolated from the Cynoglossus semilaevis culture tank in a recirculating mariculture system. Int. J. Syst. Evol. Microbiol. 64, 3926–3930. 10.1099/ijs.0.063305-0 25201915

[B44] Lombard V. Golaconda Ramulu H. Drula E. Coutinho P. M. Henrissat B. (2014). The carbohydrate-active enzymes database (CAZy) in 2013. Nucleic Acids Res. 42, D490–D495. 10.1093/nar/gkt1178 24270786 PMC3965031

[B45] Lucas-Elió P. Marco-Noales E. Espinosa E. Ordax M. López M. M. Garcías-Bonet N.. (2011). Marinomonas alcarazii sp. nov., M. rhizomae sp. nov., M. foliarum sp. nov., M. posidonica sp. nov. and M. aquiplantarum sp. nov., isolated from the microbiota of the seagrass Posidonia oceanica. Int. J. Syst. Evol. Microbiol. 61, 2191–2196. 10.1099/ijs.0.027227-0 20935088

[B46] Macián M. C. Arahal D. R. Garay E. Pujalte M. J. (2005). Marinomonas aquamarina sp. nov., isolated from oysters and seawater. Syst. Appl. Microbiol. 28, 145–150. 10.1016/j.syapm.2004.12.003 15830807

[B47] Mann A. J. Hahnke R. L. Huang S. Werner J. Xing P. Barbeyron T.. (2013). The genome of the alga-associated marine flavobacterium Formosa agariphila KMM 3901T reveals a broad potential for degradation of algal polysaccharides. Appl. Environ. Microbiol. 79, 6813–6822. 10.1128/AEM.01937-13 23995932 PMC3811500

[B48] Martin M. Biver S. Steels S. Barbeyron T. Jam M. Portetelle D.. (2014a). Identification and characterization of a halotolerant, cold-active marine endo-beta-1,4-glucanase by using functional metagenomics of seaweed-associated microbiota. Appl. Environ. Microbiol. 80, 4958–4967. 10.1128/AEM.01194-14 24907332 PMC4135742

[B49] Martin M. Portetelle D. Michel G. Vandenbol M. (2014b). Microorganisms living on macroalgae: diversity, interactions, and biotechnological applications. Appl. Microbiol. Biotechnol. 98, 2917–2935. 10.1007/s00253-014-5557-2 24562178

[B50] Marzinelli E. M. Campbell A. H. Zozaya Valdes E. Vergés A. Nielsen S. Wernberg T.. (2015). Continental-scale variation in seaweed host-associated bacterial communities is a function of host condition, not geography. Environ. Microbiol. 17, 4078–4088. 10.1111/1462-2920.12972 26148974

[B51] Matsuo Y. Suzuki M. Kasai H. Shizuri Y. Harayama S. (2003). Isolation and phylogenetic characterization of bacteria capable of inducing differentiation in the green alga Monostroma oxyspermum. Environ. Microbiol. 5, 25–35. 10.1046/j.1462-2920.2003.00382.x 12542710

[B52] Michel G. Chantalat L. Duee E. Barbeyron T. Henrissat B. Kloareg B.. (2001). The kappa-carrageenase of P. carrageenovora features a tunnel-shaped active site: a novel insight in the evolution of Clan-B glycoside hydrolases. Structure 9, 513–525. 10.1016/S0969-2126(01)00612-8 11435116

[B53] Michel G. Czjzek M. (2013). Polysaccharide-degrading enzymes from marine bacteria, in Marine Enzymes for Biocatalysis:Sources, Biocatalytic Characteristic and Bioprocesses of Marine Enzymes, ed Trincone A. (Cambridge: Woodhead Publishing Limited), 429–464.

[B54] Nadkarni M. A. Martin F. E. Jacques N. A. Hunter N. (2002). Determination of bacterial load by real-time PCR using a broad-range (universal) probe and primers set. Microbiology 148, 257–266. 10.1099/00221287-148-1-257 11782518

[B55] Nedashkovskaya O. I. (2006). Formosa agariphila sp. nov., a budding bacterium of the family *Flavobacteriaceae* isolated from marine environments, and emended description of the genus Formosa. Int. J. Syst. Evol. Microbiol. 56, 161–167. 10.1099/ijs.0.63875-0 16403882

[B56] Nedashkovskaya O. I. Suzuki M. Vancanneyt M. Cleenwerck I. Lysenko A. M. Mikhailov V. V.. (2004). Zobellia amurskyensis sp. nov., Zobellia laminariae sp. nov. and Zobellia russellii sp. nov., novel marine bacteria of the family *Flavobacteriaceae*. Int. J. Syst. Evol. Microbiol. 54, 1643–1648. 10.1099/ijs.0.63091-0 15388723

[B57] Neumann A. M. Balmonte J. P. Berger M. Giebel H.-A. Arnosti C. Voget S.. (2015). Different utilization of alginate and other algal polysaccharides by marine *Alteromonas macleodii* ecotypes. Environ. Microbiol. 17, 3857–3868. 10.1111/1462-2920.12862 25847866

[B58] Oksanen J. Kindt R. Legendre P. O'Hara B. Simpson G. L. Solymos P. (2008). The Vegan Package. Community Ecology package. 1–174. Available online at: http://vegan.r-forge.r-project.org/

[B59] Olsen J. L. Zechman F. W. Hoarau G. Coyer J. A. Stam W. T. Valero M. (2010). The phylogeographic architecture of the fucoid seaweed Ascophyllum nodosum: an intertidal “marine tree” and survivor of more than one glacial-interglacial cycle. J. Biogeogr. 37, 842–856. 10.1111/j.1365-2699.2009.02262.x

[B60] Park S. C. Hwang Y. M. Lee J. H. Baik K. S. Seong C. N. (2013). Algibacter agarivorans sp. nov. and Algibacter agarilyticus sp. nov., isolated from seawater, reclassification of Marinivirga aestuarii as Algibacter aestuarii comb. nov. and emended description of the genus Algibacter. Int. J. Syst. Evol. Microbiol. 63, 3494–3500. 10.1099/ijs.0.051300-0 23543504

[B61] Park S. Oh K. H. Lee S. Y. Oh T. K. Yoon J. H. (2012). Cellulophaga geojensis sp. nov., a member of the family *Flavobacteriaceae* isolated from marine sand. Int. J. Syst. Evol. Microbiol. 62, 1354–1358. 10.1099/ijs.0.033340-0 21828016

[B62] Popper Z. A Michel G. Hervé C. Domozych D. S. Willats W. G. T. Tuohy M. G.. (2011). Evolution and diversity of plant cell walls: from algae to flowering plants. Annu. Rev. Plant Biol. 62, 567–590. 10.1146/annurev-arplant-042110-103809 21351878

[B63] Potin P. Bouarab K. Salaün J.-P. Pohnert G. Kloareg B. (2002). Biotic interactions of marine algae. Curr. Opin. Plant Biol. 5, 308–317. 10.1016/S1369-5266(02)00273-X 12179964

[B64] Rebuffet E. Groisillier A. Thompson A. Jeudy A. Barbeyron T. Czjzek M.. (2011). Discovery and structural characterization of a novel glycosidase family of marine origin. Environ. Microbiol. 13, 1253–1270. 10.1111/j.1462-2920.2011.02426.x 21332624

[B65] Romanenko L. A. Zhukova N. V. Rohde M. Lysenko A. M. Mikhailov V. V. Stackebrandt E. (2003). Glaciecola mesophila sp. nov., a novel marine agar-digesting bacterium. Int. J. Syst. Evol. Microbiol. 53, 647–651. 10.1099/ijs.0.02469-0 12807181

[B66] Saitou N. Nei M. (1987). The neighbor-joining method: a new method for reconstructing phylogenetic trees. Mol. Biol. Evol. 4, 406–425. 3447015 10.1093/oxfordjournals.molbev.a040454

[B67] Stackebrandt E. (2011). Molecular taxonomic parameters. Microbiol. Aust. 32, 59–61. Available online at: http://microbiology.publish.csiro.au/?paper=MA11059

[B68] Stratil S. B. Neulinger S. C. Knecht H. Friedrichs A. K. Wahl M. (2013). Temperature-driven shifts in the epibiotic bacterial community composition of the brown macroalga Fucus vesiculosus. Microbiologyopen 2, 338–349. 10.1002/mbo3.79 23568841 PMC3633357

[B69] Stratil S. B. Neulinger S. C. Knecht H. Friedrichs A. K. Wahl M. (2014). Salinity affects compositional traits of epibacterial communities on the brown macroalga Fucus vesiculosus. FEMS Microbiol. Ecol. 88, 272–279. 10.1111/1574-6941.12292 24490649

[B70] Stroobants A. Degrune F. Olivier C. Muys C. Roisin C. Colinet G.. (2014). Diversity of bacterial communities in a profile of a winter wheat field: known and unknown members. Microb. Ecol. 68, 822–833. 10.1007/s00248-014-0458-6 25008985

[B71] Sugano Y. Matsumoto T. Kodama H. Noma M. (1993). Cloning and sequencing of agaa, a unique agarase 0107 gene from a marine bacterium, vibrio sp. strain JT0107. Appl. Environ. Microbiol. 59, 3750–3756. 8285681 10.1128/aem.59.11.3750-3756.1993 PMC182527

[B72] Tanaka R. Shibata T. Miyake H. Mori T. Tamaru Y. Ueda M. (2015). Temporal fluctuation in the abundance of alginate-degrading bacteria in the gut of abalone *Haliotis gigantea* over 1 year. Aquac. Res. [Epub ahead of print]. Available online at: http://onlinelibrary.wiley.com/doi/10.1111/are.12740/references 10.1111/are.12740

[B73] Tindall B. J. Rosselló-Móra R. Busse H. J. Ludwig W. Kämpfer P. (2010). Notes on the characterization of prokaryote strains for taxonomic purposes. Int. J. Syst. Evol. Microbiol. 60, 249–266. 10.1099/ijs.0.016949-0 19700448

[B74] Venter J. C. Remington K. Heidelberg J. F. Halpern A. L. Rusch D. Eisen J. A.. (2004). Environmental genome shotgun sequencing of the Sargasso Sea. Science 304, 66–74. 10.1126/science.1093857 15001713

[B75] Wang F.-Q. Lin X.-Z. Chen G.-J. Du Z.-J. (2015). Colwellia arctica sp. nov., isolated from Arctic marine sediment. Antonie Van Leeuwenhoek 107, 723–729. 10.1007/s10482-014-0366-2 25601047

[B76] Wang L. Li S. Yu W. Gong Q. (2014). Cloning, overexpression and characterization of a new oligoalginate lyase from a marine bacterium, Shewanella sp. Biotechnol. Lett. 37, 665–671. 10.1007/s10529-014-1706-z 25335746

[B77] Wiese J. Thiel V. Nagel K. Staufenberger T. Imhoff J. F. (2009). Diversity of antibiotic-active bacteria associated with the brown alga Laminaria saccharina from the Baltic Sea. Mar. Biotechnol. (NY). 11, 287–300. 10.1007/s10126-008-9143-4 18855068

[B78] Yao Z. Wang F. Gao Z. Jin L. Wu H. (2013). Characterization of a κ-carrageenase from marine cellulophaga lytica strain n5-2 and analysis of its degradation products. Int. J. Mol. Sci. 14, 24592–24602. 10.3390/ijms141224592 24351836 PMC3876130

[B79] Yong J. J. Park S. J. Kim H. J. Rhee S. K. (2007). Glaciecola agarilytica sp. nov., an agar-digesting marine bacterium from the East Sea, Korea. Int. J. Syst. Evol. Microbiol. 57, 951–953. 10.1099/ijs.0.64723-0 17473239

[B80] Yooseph S. Sutton G. Rusch D. B. Halpern A. L. Williamson S. J. Remington K.. (2007). The Sorcerer II global ocean sampling expedition: expanding the universe of protein families. PLoS Biol. 5:e16. 10.1371/journal.pbio.0050016 17355171 PMC1821046

[B81] Yung P. Y. Burke C. Lewis M. Kjelleberg S. Thomas T. (2011). Novel antibacterial proteins from the microbial communities associated with the sponge Cymbastela concentrica and the green alga Ulva australis. Appl. Environ. Microbiol. 77, 1512–1515. 10.1128/AEM.02038-10 21183639 PMC3067216

