# Supplementary material for: The Cultivable Surface Microbiota of the Brown Alga Ascophyllum nodosum is Enriched in Macroalgal-Polysaccharide-Degrading Bacteria
Source: Front Microbiol. 2015 Dec 24;6:1487. doi: 10.3389/fmicb.2015.01487 (PMC4690005; doi:10.3389/fmicb.2015.01487)
Supplement: Table S1 — Percentage ranges and averages of the query coverages and identity percentages for the alignments with the 291 V3-V4 region sequences of the isolates to which a genus have been attributed. [file Table1.DOCX]

|  | **Query coverage** | | | | **Identity %** | | |
| --- | --- | --- | --- | --- | --- | --- | --- |
|  | **<90%** | **90-97%** | **>97%** | **Average** | **<97%** | **>97%** | **Average** |
| **Sample 1** | 1 | 11 | 84 | **98.1%** | 5 | 90 | **98.6%** |
|  | 1% | 11% | 88% |  | 5% | 94% |  |
| **Sample 2** | 14 | 7 | 74 | **94.5%** | 3 | 93 | **99.0%** |
|  | 15% | 7% | 77% |  | 3% | 97% |  |
| **Sample 3** | 1 | 4 | 94 | **98.6%** | 4 | 95 | **99.0%** |
|  | 1% | 4% | 95% |  | 4% | 96% |  |
| **Total** | 16 | 22 | 252 | **97.2%** | 12 | 278 | **98.9%** |
|  | 5% | 8% | 87% |  | 4% | 96% |  |

**Table S1: Percentage ranges and averages of the query coverages and identity percentages for the alignments with the 291 V3-V4 region sequences of the isolates to which a genus have been attributed.**. Average query coverages and identity percentages for each sample and for the total isolated microbiota are indicated in bold.
