# Supplementary figures and images for: The Cultivable Surface Microbiota of the Brown Alga Ascophyllum nodosum is Enriched in Macroalgal-Polysaccharide-Degrading Bacteria
Source: Front Microbiol. 2015 Dec 24;6:1487. doi: 10.3389/fmicb.2015.01487 (PMC4690005; doi:10.3389/fmicb.2015.01487)

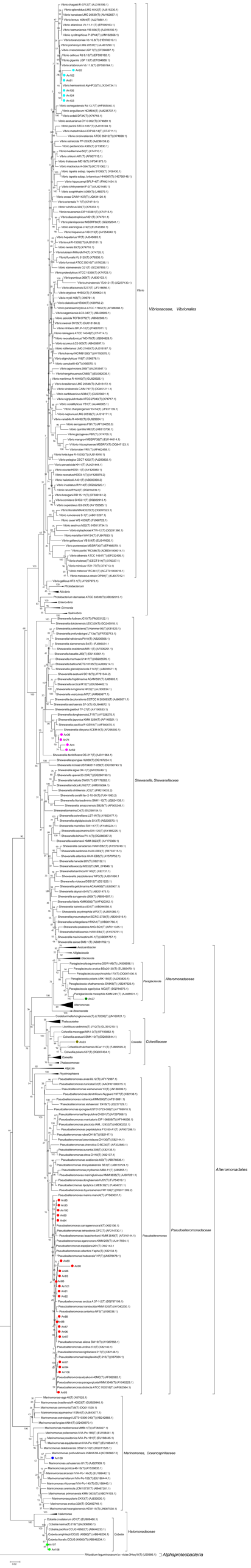

Supplement: Figure S1 — Detailed phylogenetic tree for the Gammaproteobacteria. [file Image1.PDF]
